# *Vibrio mimicus* wound infection in a burn patient

**DOI:** 10.1016/j.radcr.2021.03.021

**Published:** 2021-04-09

**Authors:** Anne Yang, Mohamed Yassin, Tung Phan

**Affiliations:** a Department of Medicine, University of Pittsburgh Medical Center, Pittsburgh, Pennsylvania, USA; b Division of Clinical Microbiology, University of Pittsburgh and University of Pittsburgh Medical Center, Pittssburgh, Pennsylvania, USA

**Keywords:** Burn, trauma, *V mimicus*, polymicrobial infection

## Abstract

Burns are one of the most common and devastating forms of trauma. Burns are a significant problem with high associated morbidity and mortality worldwide. Burn wound infection is a serious complication, which plays an important role in increasing the overall fatality rate in burn patients. In this study, we report a case of the polymicrobial burn wound infection involving *V mimicus* in a 56-year-old male, who was transferred from an outside hospital to the inpatient burn unit after sustaining traumatic and burn injuries in a firework explosion accident. The patient underwent surgical treatment and antibiotics with good improvement. Although rare, our case study will help to underscore the important role of *V mimicus* as a human pathogen.

## Introduction

Burns are the fourth most common type of trauma worldwide and contribute significantly to the burden of death and disability [1]. Burns are a significant public health problem, accounting for an estimated 180,000 deaths per year worldwide. In the United States, there are more than 500,000 people requiring medical treatment, 40,000 hospitalizations, and 4000 deaths per year [2]. The direct cost of medically treated burns may exceed $1 billion, not including the indirect costs of disability and rehabilitation [3]. Burn wound infection is a serious complication, which plays an important role in increasing the overall fatality rate in burn patients [4,5]. Before the introduction of penicillin G in the early 1950s, *Streptococcus pyogenes* was the predominant pathogen implicated in burn wound infections and was a significant cause of death in severely burned patients [4]. After that, *Staphylococcus aureus* becomes a common cause of early burn wound infections [4]. Gram-negative microorganisms have long been known to cause serious infections in burn patients. The majority (60.2%) of burn wound infections were due to gram-negative microorganisms [6]. *Pseudomonas aeruginosa* represents a major concern in burn patient care settings [7,8]. Here we report a case of *V mimicus* soft tissue infection in a burn patient with multiple traumatic injuries.

## Case presentation

**Fig. 1:**
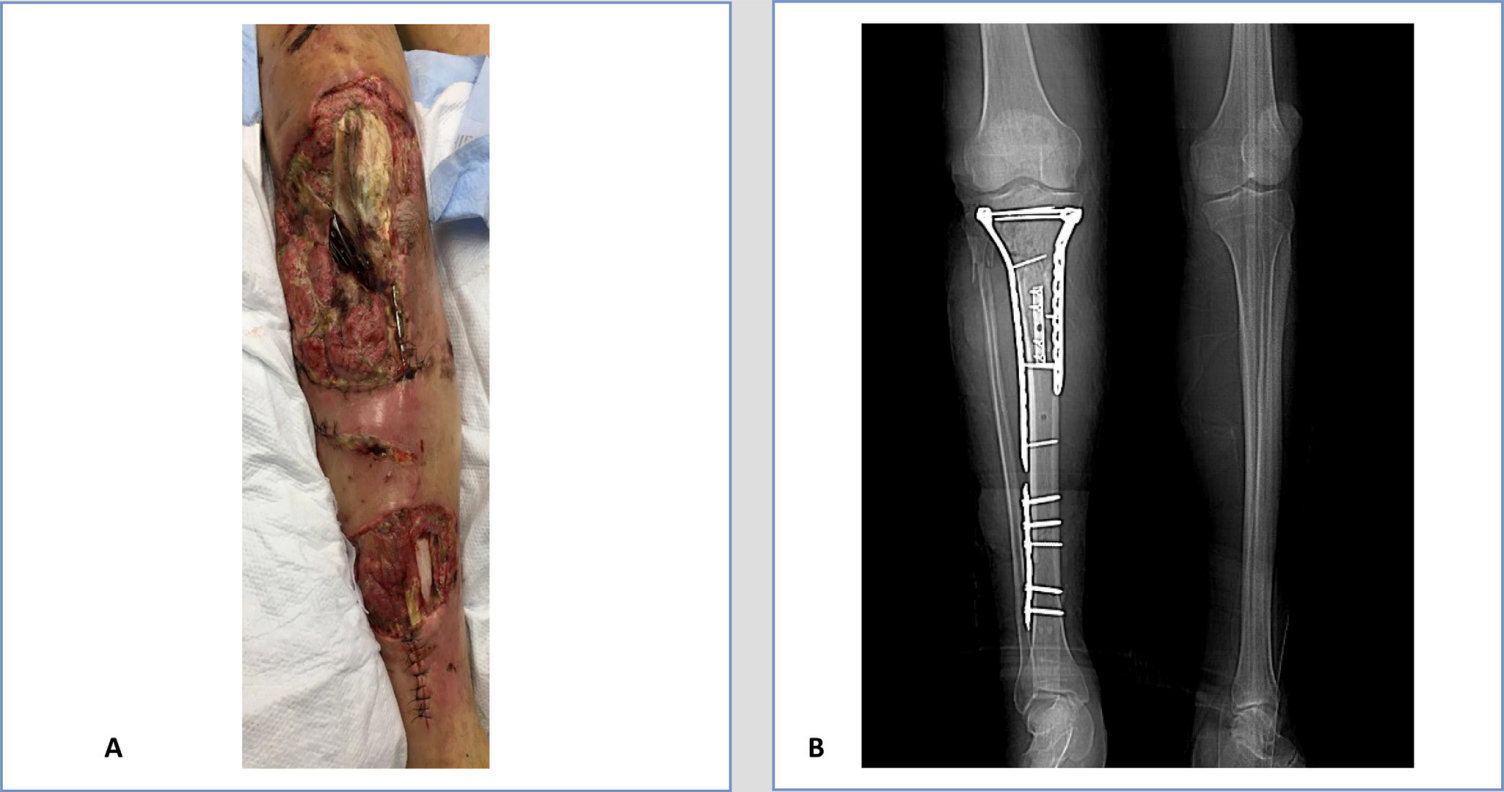
(**A**). The clinical photograph and (**B**) the radiograph of the right open tibial-fibular fracture status post open reduction internal fixation (ORIF) with overlying wound and exposed hardware at day 7 after the firework explosion accident.

A 56-year-old male with a medical history of polysubstance abuse and hypertension was transferred from an outside hospital to the inpatient burn unit after sustaining traumatic and burn injuries in a firework explosion accident one week prior. Upon admission, he was afebrile and hemodynamically stable (blood pressure 127/82 mm Hg, heart rate 99 bpm, respiratory rate 16/min, and blood oxygen saturation level 96% on room air). His complicated injury complex included first and second-degree burns to the face, right upper, and right lower extremities; right deltoid wound with exposed humerus suggest picture reference of the abdominal wall, leg and shoulder; right lower extremity open wounds status post debridement now with vacuum-assisted closures; foreign objects in the scrotum, left medial thigh, and medial forearm status post scrotal repair with staples; and, a right open tibial-fibular fracture status post open reduction internal fixation (ORIF) with overlying wound and exposed hardware (Fig. 1). He had also undergone an exploratory laparotomy at the outside hospital. As the only culture data from the outside hospital yielded no growth, he was started on broad-spectrum coverage with vancomycin, cefepime, and metronidazole. He had daily hydrotherapy for his partial-thickness burns. On day 2, surgical debridement of his right shoulder and right leg was performed, with right leg muscle necrosis and purulent drainage visualized. Aerobic wound cultures of the right leg grew pan-sensitive *V mimicus* and *Aeromonas hydrophilia*, and the antibiotic regimen was narrowed to cefepime only. Vancomycin and metronidazole were discontinued. After wound cultures from a secondary surgical debridement of the right leg again grew *V mimicus* and now *Pseudomonas stutzeri*, the patient elected to undergo a right above-knee amputation for source control of acute osteomyelitis and soft tissue necrosis. Subsequent wound cultures from above the right knee amputation site grew *Candida albicans*. The patient was started on fluconazole as there were operative plans for a right latissimus dorsi flap. He was eventually discharged to a skilled nursing facility on fluconazole and cefepime for six weeks total from when he first cleared his wound cultures. This patient was fitted with a prothesis and made a full recovery with good return of functional performance status post-amputation.

## Discussion

**Fig. 2:**
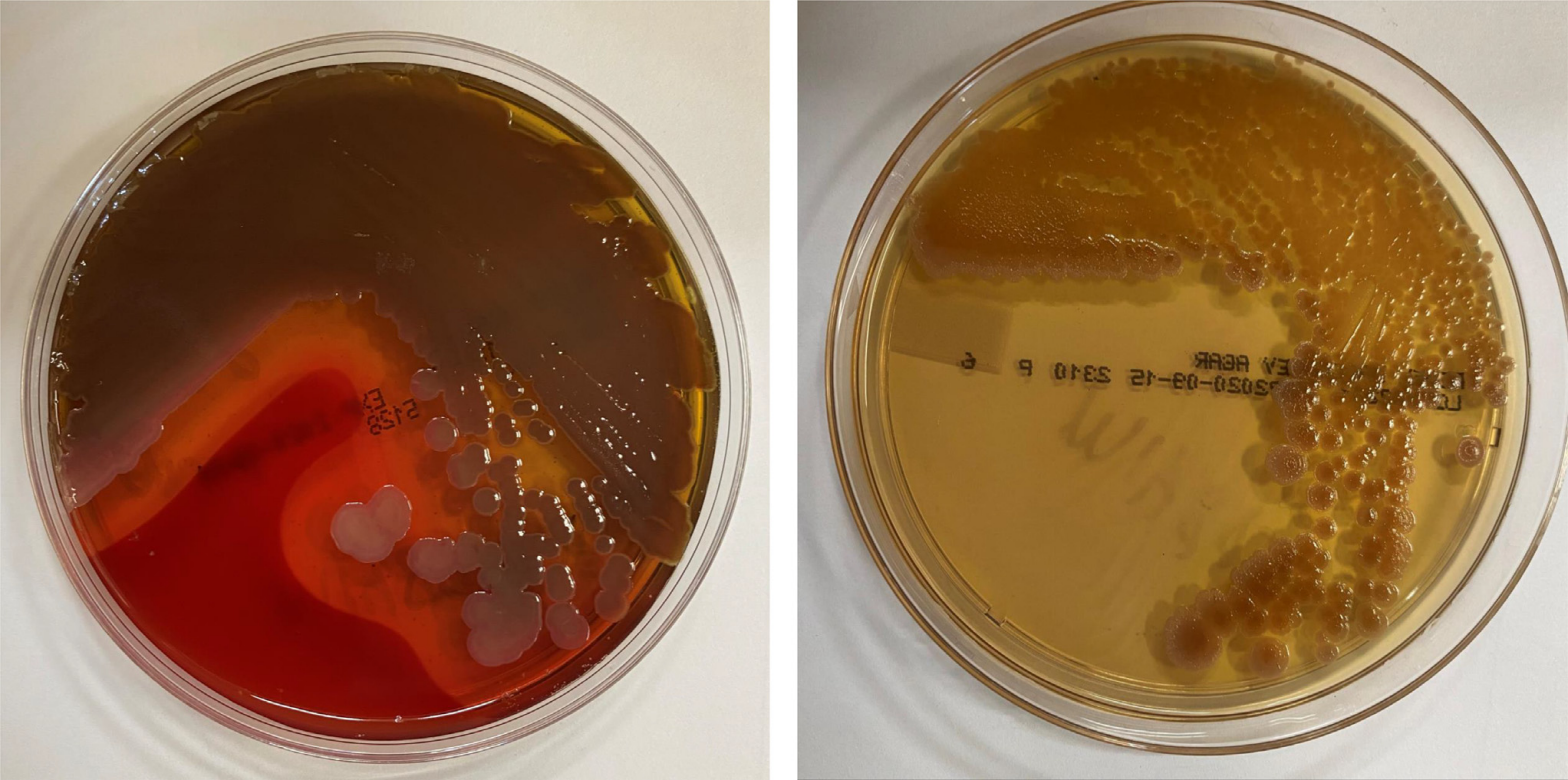
*V mimicus* grew on sheep blood agar and MacConkey agar. *V mimicus* is beta hemolytic on sheep blood agar and non-lactose fermenting on MacConkey agar.

*Vibrio* species are water microorganisms commonly found in marine or brackish water environments. Among currently described *Vibrio* species, *Vibrio cholerae, Vibrio vulnificus*, and *Vibrio parahaemolyticus* are most frequently associated with human illnesses [9,10]. Human illnesses in two major groups: cholera and non-cholera infections. *V cholerae* is the etiological agent of cholera, a severe diarrhea. Non-cholera *Vibrio* species such as *V vulnificus*, and *V parahaemolyticus*, cause vibriosis, a group of infections with different clinical manifestations depending on the pathogen species, route of infection, and host susceptibility [10]. *V mimicus* was identified in 1983, and it has been recognized as an uncommon cause of gastroenteritis occurring after recent ingestion of seafood and in acute otitis media after exposure to seawater [11]. Though found primarily in marine ecosystems, some species like *V mimicus* are nonhalophilic and can live in fresh water. A large foodborne outbreak caused by *V mimicus* involving 306 people was related to the ingestion of dishes containing freshwater fish and seafood in Chiang Mai, Thailand [12]. A cluster of severe diarrhea caused by *V mimicus* infection among four persons associated with crayfish consumption was reported in Washington State, USA [13]. In this case, the tissue specimens collected from this patient during the surgery were submitted to the clinical microbiology laboratory for fungal and bacterial cultures. *V mimicus* was identified by Vitek 2, which is a fully automated system that performs bacterial identification using fluorescence-based technology. Microscopic examination of a gram-stained smear confirmed that this bacterium is gram negative bacillus. On sheep blood agar, *V mimicus* is beta hemolytic, and on MacConkey agar is non-lactose fermenting (Fig. 2). Polymicrobial burn wound infection is often seen, and it is increasingly being reported in burn patients [14] as seen in our case. However, the burn wound infection associated with V mimicus is rare, and our keyword (*V mimicus* and wound) search in PubMed did not identify any other previously reported cases. Burn infections can occur at the healthcare facility, but commonly at the time of burn. Environmental pathogens as fungi and water-borne bacteria are highly likely to occur at the burn environment even if it took days or weeks to manifest. The patient's infection was likely acquired at the burn site due to contamination of burn with stream water. The choice of ORIF in a burn heavily contaminated wound is likely suboptimal. Open well-drained wounds with external fixators as needed is likely a better option for avoiding aggressive severe infections as *Vibrio* species.

In summary, we present the case of burn wound infection associated with *V mimicus* in a burn patient with multiple traumatic injuries. Although rare, our case study will help to underscore the important role of *V mimicus* as a human pathogen.

## Author contributions

TP, MY and AY: designed the study and wrote the manuscript.

## Ethical approval

Approval from the ethical committee was not required due to the nature of this case report. Abiding by the Declaration of Helsinki, patient anonymity was guaranteed.
